# Distal Tibiofibular Syndesmosis: Anatomy, Biomechanics, Injury and Management

**DOI:** 10.2174/1874325001711010670

**Published:** 2017-07-31

**Authors:** Chi Pan Yuen, Tun Hing Lui

**Affiliations:** 1 Department of Orthopaedics and Traumatology, Li Ka Shing Faculty of Medicine, The University of Hong Kong, Hong Kong, China; 2 Department of Orthopaedics and Traumatology, North District Hospital, 9 Po Kin Road, Sheung Shui, NT, Hong Kong, China

**Keywords:** Distal tibiofibular syndesmosis, Anatomy, Biomechanics, Injury, Ankle joint, Ligament complex, Function, Ankle arthroscopy

## Abstract

A stable and precise articulation of the distal tibiofibular syndesmosis is essential for normal motion of the ankle joint. Injury to the syndesmosis occurs through rupture or bony avulsion of the syndesmotic ligament complex. External rotation of the talus has been identified as the major mechanism of syndesmotic injury. None of the syndesmotic stress tests was sensitive or specific; therefore the diagnosis of syndesmotic injury should not be made based on the medical history and physical examination alone. With the improvement in ankle arthroscopic technique, it can be used as a diagnostic and therapeutic tool in the management of distal tibiofibular syndesmosis injury.

## INTRODUCTION

A syndesmosis is defined as a fibrous joint in which two adjacent bones are linked by a strong membrane or ligaments. For distal tibiofibular syndesmosis, its chief function is to maintain the congruency of the tibiotalar interface under physiologic axial loads. In order to recognize the function, biomechanics, injury mechanism and management of distal tibiofibular syndesmotic injury, a detailed understanding of the bony and soft tissue anatomy is essential.

## ANATOMY

For the bony anatomy, the medial rough convex surface of the distal fibula articulate with the lateral triangular fibular notch of the distal tibia to form a fibrous joint, which is linked by strong ligaments.

The tibial part of the syndesmosis forms a concave triangle. The apex located around 6-8cm above the level of the talocrural joint and the lateral ridge of the tibia bifurcates caudally [1]. The antero-lateral margin forms the anterior tubercle (Chaput’s tubercle) and the posterolateral margin becomes the posterior tubercle [2]. Anterior tubercle is larger thus prevents forward slipping of the fibula. Posterior tubercle functions as a fulcrum during external rotation injury and the fibula spins around its longitudinal axis.

The fibular part forms a convex triangle. The apex is located at the same level as the tibial triangle. It also bifurcates into an anterior tubercle (Wagstaffe–Le Fort tubercle) and an insignificant posterior tubercle. The base of the triangle is located just above the articular facet of the lateral malleolus.

At the base of the syndesmosis, there is a small area where the tibia and fibula are in direct contact, which is called the tibiofibular contact zone. Its facets are covered with a thin layer of hyaline cartilage. Its size varies and may be absent in healthy subject [1, 3, 4]. Although its function is unclear, this articulation can act as a guide for the alignment of the ankle mortise in ankle fracture fixation or syndesmosis reconstruction [2].

For the soft tissue, 4 ligaments form the syndesmotic ligament complex: anterior inferior tibiofibular ligament (AITFL), posterior inferior tibiofibular ligament (PITFL), tibiofibular interosseous ligament (TFIL) and transverse tibiofibular ligament (TTFL). These ligaments work together with the bony restrain to maintain the integrity between the distal tibia and the fibula by resisting the axial, rotational and translational forces. There is also a synovial-lined plica extends from the tibiotalar joint called syndesmotic recess. It attached to the distal tibia medially and the distal fibula laterally with an interposing fat-containing synovial fold [3, 5]. In acute syndesmotic injury, this recess can tear causing leakage of contrast into the incisura tibialis [6]. In chronic syndesmotic injury, the synovial lining may become irregular due to inflammation [7].

AITFL run from anterior tubercle of the distal tibia to the anterior tubercle of the distal fibular, in an oblique way from proximal-medially to distal-laterally. It composed of three bundles forming a trapezoidal shape. Microscopically the ligament has a multi-fascicular pattern with interposing fatty tissue [3, 4]. Occasionally, an accessory antero-inferior tibiofibular ligament, the Bassett’s ligament, runs inferior and parallel to the AITFL. This is a potential cause of antero-lateral ankle impingement in case of synovitis or scarring. As 20% of the AITFL is intra-articular [8], the deepest fibre can be seen during ankle arthroscopy. AITFL is the weakest of the four syndesmotic ligaments and is the first ligament subjected to stress upon an external rotation force of the fibula around its longitudinal axis [1].

PITFL extends from the posterior tibial malleolus to the posterior tubercle of the fibula and runs in a proximal-medial to distal-lateral manner. This forms a triangular shape with a broad base at the tibial insertion [4]. Microscopically it has also a multi-fascicular pattern. Tear of the PITFL can occur in Lauge-Hansen supination-eversion, pronation-eversion or pronation-abduction injury. However, as the ligament itself is strong and thick, force usually resulted in a posterior malleolus avulsion fracture rather than pure ligamentous tear [9].

TFIL is formed by condensation and thickening of the lowermost end of the interosseous membrane. Microscopically fatty tissue and steep running fascicles were arranged into a spatial network of pyramidal shape [2]. This ligament functions as a “spring” to accommodate slight separation of the mortise during ankle dorsiflexion and the force generated during heel strike.

TTFL runs horizontally between the proximal margin of the fibular malleolar fossa and the dorso-distal rim of the tibia distal to the PITF, it may extend to as far as the dorsal aspect of the medial malleolus [4]. It is a thick and roundish ligament functions as a labrum analogue to deepen the postero-inferior rim of the tibia. However, whether the transverse ligament and the most distal part of PITFL are two distinct structures is still controversial [10, 11]. Another controversy is that whether the intermalleolar ligament (IML), the coalescence of the fibres from PITFL and TL, exist or not. The occurrence of IML varies from 19% in MRI to 82% in dissected anatomical specimens [10, 12, 13]. Some authors proposed this ligament can cause posterior impingement syndrome especially in ballet dancers [13].

Functionally, anatomical study suggested that the relative importance of individual syndesmotic ligaments to the overall syndesmotic stability was: 35% by the AITFL, 33% by the TTFL, 22% by the TFIL and 9% by the PITFL [14]

## BIOMECHANICS

By using radiostereometry technique, the normal kinematics of the tibiofibular syndesmosis during weight bearing and external rotation stress were analyzed in normal subjects [15]. Only very small rotations and displacements were detected indicating the fibula is closely attached to the tibia. During the external rotation stress test with a 75 Nm force, it caused 2 - 5° external rotation, 0-2.5 mm medial translation and 1.0-3.1 mm posterior displacement of the fibula. While moving from plantar flexion to dorsiflexion, Peter *et al.* reported a 1.25mm lateral translation and 2° external rotation of the lateral malleolus [16]. These biomechanical studies proved that distal tibiofibular syndesmosis is highly stable.

Close *et al.* reported that when the deep horizontal section of the deltoid ligament is cut, this diastasis increases to 3.7 mm [17, 18]. Ramsey and Hamilton [19] described when the talus moves laterally by 1 mm, the contact area in the tibiotalar articulation is decreased by 42%. Furthermore, Burns *et al.* [20] have shown that a complete disruption of syndesmosis with a disruption of the deltoid ligament causes a 40% decrease in the tibiotalar contact area and a 36% increase in the tibiotalar contact pressures. Therefore, a stable and precise articulation is essential for normal motion of the ankle joint.

## INJURY MECHANISM

Injury to the syndesmosis occurs through rupture or bony avulsion of the syndesmotic ligament complex [21, 22]. It was estimated that syndesmotic injury occurs in 1–11% of all ankle sprains [23], with a higher incidence of 17-74% in certain type of sports activities, *e.g.* skiers, football and hockey players [24]. Syndesmotic disruption usually takes longer to heal than common lateral ligamentous injury of the ankle [23, 24], while 40% of patients still have ankle instability symptoms 6 months after the injury. In some patients, the ligament complex failed to heal completely and resulted in a prolonged disability [14, 25].

External rotation of the talus has been identified as the major mechanism of syndesmotic injury [14, 23-25]. During external rotation of the foot the fibula is translated posteriorly and rotated externally, resulted in high tensile force acting on the AITFL. Syndesmotic injury was usually associated with ankle fracture, most commonly Weber C type [26]. However cases of distal tibiofibular dislocations with intact fibula have been reported in the past [27]. All involved a twisting load with traumatic supination injury [24]. These cases were usually missed without high index of suspicious.

Patient usually complained of pain during activity, a feeling of instability and weakness of the ankle. Furthermore, and ecchymosis at the level of the syndesmosis, are some classical signs [24, 28-30].

## CLASSIFICATION

Syndesmotic injuries are traditionally graded from I to III in the same manner as lateral ankle sprains. Grade I refers to mild stretching, grade II represents incomplete tear and grade III is a complete disruption of the syndesmosis [31]. Lui suggested a new anatomic classification system for syndesmosis diastasis [32]. The diastasis is divided into frank or occult, which further subdivided into coronal, sagittal, rotational or longitudinal pattern. The planes of instability are not mutually exclusive and play a role in describing the syndesmosis diastasis and aid in reduction.

## INVESTIGATION

Several specific syndesmosis stress tests have been described.

- The squeeze test [23], which is performed by compressing the fibula against the tibia at the midpoint of the calf. When positive, proximal compression produces distal pain around the distal tibiofibular joint.
- External rotation stress test as described by Boytim [24], external rotation force is tenderness over the AITFL, swellingapplied to the ankle in a plantigrade position with the knee flexed 90°. A positive test is noted when pain in the area of the distal tibiofibular joint is felt.
- Cotton test [33], the talus is ‘rocked’ from side to side in the ankle mortise by applying alternating medial and lateral stress to the talus. It is considered positive when a characteristic click felt in the ankle mortise and the patient experiences pain.

None of the syndesmotic stress tests was sensitive or specific [34, 35], therefore the diagnosis of syndesmotic injury should not be made based on the medical history and physical examination alone.

X-ray is the commonest imaging technique used in clinical practice. Three parameters are utilized: tibiofibular clear space (TFCS), medial clear space, and tibiofibular overlap. TFCS is more reliable as it does not influenced significantly by tibial rotation [36-38]. TFCS is defined as the distance between the lateral border of the posterior tibial tubercle and the medial border of the fibula measured on anterior/posterior and mortise views. It is measured 1 cm proximal to the distal tibial articular surface. A distance of less than 6 mm is normal [36]. However, due to the inter-observer variation, wide anatomic variability in the depth of the peroneal groove and the shape of the tibial tubercles, it is not reliable unless there is significant disruption of the distal tibiofibular syndesmosis [39, 40].

Computed tomography (CT) is proven to be more sensitive than X-Ray to evaluate the tibiofibular relationship [41]. However it is difficult to measure a 1-mm diastasis [42]. To improve the sensitivity of CT scan imaging technique, Taser *et al.* [43] rendered the three-dimensional CT data of joint space and calculated the volume of tibiofibular joint space. This enhanced the ability of the CT-scan data to detect even a 1-mm diastasis. Besides, external rotation of the distal fibula is not easily recognized in CT due to the roundish shape of the fibula at the syndesmosis level [44].

Magnetic resonance imaging has added benefit to assess the soft tissue condition directly. It has been found to be useful in both acute and chronic syndesmotic injuries [14, 45]. The diagnosis is based on the appearance of ligaments, distal tibiofibular joint congruity and the height of the tibiofibular recess [46].

As AITFL is a superficial structure, it can be well visualized with ultrasound. Milz *et al.* reported an accuracy of 85% in differentiating intact and injured ligament [47].

Most orthopedic and trauma surgeons use the well-known intraoperative “hook” test or external rotation stress test under fluoroscopic control to assess syndesmotic stability [48-50]. However, it is difficult to standardize and interpret this test.

With the improvement in ankle arthroscopic technique, it can be used as a diagnostic and therapeutic tool. It has been reported that the diagnosis of syndesmosis disruption can be made by ankle arthroscopy with a 100% accuracy [51]. Under arthroscopy, the torn parts of the anterior syndesmotic ligament can often be seen. By inserting a 3mm probe into the distal tibiofibular joint, easy turning of the transverse end around its long axis in the syndesmosis are mentioned as ways to assess the syndesmotic integrity [3, 52]. Lui suggested dynamic maneuvers during ankle arthroscopy to assess the coronal, sagittal and rotational planes stability. This can also help in syndesmosis reduction [32]. In chronic cases, although the diastasis and instability is not a constant finding but arthroscopic evaluation is helpful to detect syndesmotic ligament hypertrophy [53].

## MANAGEMENT PRINCIPLE

The general principle is to restore the ankle joint congruency and maintain the distal tibiofibular syndesmosis stability. Ideally, the implant should stabilize the syndesmosis but allow physiologic micro-motion and early mobilization, but it is not easy to achieve such goals.

Despite the numerous biomechanical, cadaveric and clinical studies concerning ankle fractures and syndesmotic injury, there is no common management consensus yet. Controversies existed from pre-operative indication, intra-operative surgical technique to post-operative rehabilitation.

Pre-operatively, there is no consensus whether an additional syndesmotic screw is indicated for specific injury patterns and fracture types [54, 55]. A transverse syndesmotic screw can effectively transfix the tibia to fibula. However screw fixation also affects the physiologic normality of the joint, leading to decreased magnitude of motion at the lower extremes of the tibia and fibula, reduced contact forces between bones, and increased stress on the crural interosseous membrane. With this concern, some authors suggested using an endobutton and transosseous suture to provide a semi-rigid dynamic stabilization of the syndesmosis [56-58].

Intra-operatively, how to do the traditional syndesmotic screw fixation is also quite controversial. For the level of screw fixation, there are expert opinions suggesting from 2 to 5cm above the joint line. One review article concluded that there is no difference in outcome for both supra- or trans-syndesmotic placement of the syndesmotic screw, provided that it can obtain the most stable construct [59]. Concerning the ankle position during screw fixation, the original idea was proposed by Olerud [60]. In cadaveric specimen, the dorsiflexion range decreased by an average of 0.1° for every degree of increase in plantar flexion at the time of screw insertion. Therefore, the author suggested that the ankle should be placed in maximal dorsiflexion during placement of the syndesmotic screw to reduce the risk of stiffness. This concept has been challenged by other authors [61]. Nowadays, the foot is commonly maintained in plantigrade position during application of the screw [25]. Considering optimal number of cortices penetration, the biomechanical studies were contradicting [62, 63]. But clinical studies were more favorable for three cortices engagement, as the clinical outcome was comparable especially for long-term follow up. There was also lower rate of distal tibiofibular synostosis [64-67].

Post-operative rehabilitation program, for example, optimal time to weight bearing or whether the screw needed to be removed before weight bearing is also unanswered [68]. Needleman *et al.* suggested non-weight bearing until the screw has been removed 6 to 12 weeks postoperatively [69]. This is based on the concept that rigid syndesmotic fixation prohibits the normal fibular motion and mortise widening during ankle movement [70]. Riegels-Nielsen and Greiff [26] stated that premature weight bearing can cause the syndesmotic screw to loosen or break and the use of 3.5-mm screws and screws penetrating only 2 tibial cortices have a greater risk of breakage [71]. However Moore and Hamid [66, 72] stated that retention of syndesmotic screw, even with screw breakage did not affect the clinical outcome. Therefore weight bearing could be allowed at 6 to 10 weeks without routine screw removal.

After all, some studies concluded that no matter syndesmotic screws or other stabilizing techniques have all been effective in stabilizing the distal tibiofibular syndesmosis to allow ligamentous healing or fibrous union [73]. However patients who required syndesmotic stabilization in addition to ankle fracture fixation had poorer outcomes [74].

## CONCLUSION

The distal tibiofibular syndesmosis must be stable and congruent for normal ankle motion under physiological load. Syndesmotic injury usually occurred in ankle sprain with external rotation of the talus that resulted at either ligamentous rupture or bony avulsion of the syndesmotic ligament complex. Making the diagnosis purely from history and physical examination is not easy as most of the syndesmotic stress tests were not sensitive and specific. With high index of suspicion, CT imaging or ankle arthroscopy can be used in both diagnostic and therapeutic means. Syndesmosis injury should be best managed in the acute phase to restore the ankle congruency and maintain the stability. There is no consensus how should the syndesmosis be fixed in acute stage. Syndesmotic screws, endobutton or transosseous suture are all reasonable options with similar results. If the acute injury was not well managed, studies have demonstrated poor functional outcomes and the development of post-traumatic ankle arthritis related to poorly reduced or stabilized syndesmotic disruption.
